# Lipidomic alterations in human saliva from cystic fibrosis patients

**DOI:** 10.1038/s41598-022-24429-6

**Published:** 2023-01-12

**Authors:** Marianna Caterino, Roberta Fedele, Vincenzo Carnovale, Alice Castaldo, Monica Gelzo, Paola Iacotucci, Margherita Ruoppolo, Giuseppe Castaldo

**Affiliations:** 1 Department of Molecular Medicine and Medical Biotechnology, School of Medicine, University of Naples Federico II, 80131 Naples, Italy; 2 CEINGE - Biotecnologie Avanzate F. Salvatore, s.c.ar.l, 80145 Napoli, Italy; 3 Department of Translational Medical Sciences, University of Naples Federico II, Naples, Italy; 4 Department of Clinical Medicine and Surgery, University of Naples Federico II, Naples, Italy

**Keywords:** Cystic fibrosis, Lipidomics

## Abstract

Cystic fibrosis is a hereditary metabolic disorder characterized by impaired traffic of chloride ions and water through membranes of the respiratory and gastrointestinal, that causes inadequate hydration of airway surfaces, dehydrated mucous secretions and a high-sodium chloride sweat. Although the classical presentation of the condition is well known, a better characterization of metabolic alterations related is need. In particular, the metabolic composition alterations of biological fluids may be influence by the disease state and could be captured as putative signature to set targeted therapeutic strategies. A targeted comprehensive mass spectrometry-based platform was employed to dissect the lipid content of saliva samples form CF patients, in order to investigate alterations in the lipid metabolic homeostasis related to the pathology, chronic obstructive pulmonary disease, *Pseudomonas Aeruginosa* infection, pancreatic insufficiency, liver disfunction and diabetes-related complications.

## Introduction

Cystic fibrosis (CF) is a systemic autosomal recessive syndrome characterized by progressive obstructive lung disease and pancreatic insufficiency (PI). The disease is caused by recessive mutations in the *cystic fibrosis transmembrane conductance regulator* (*CFTR*) gene, which encodes an ion channel that facilitates the movement of chloride and bicarbonate ions through the transepithelial membranes as well as indirectly affects sodium currents via its interaction with the epithelial sodium channel^1^.

The mutated CFTR protein is associated to impaired traffic of chloride ions and water through membranes of the respiratory and gastrointestinal tracts, that causes inadequate hydration of airway surfaces, dehydrated mucous secretions and a high-sodium chloride sweat.

Changes in fatty acid levels were found in plasma of patients with CF mainly due to the lower fat intake also due to malabsorption^2^, even if such alterations are observed also in absence of malnutrition^3^. In fact, lipid imbalance is observed also in CF cell lines with relevant changes in phospholipids, cholesterol esters and glycosylated sphingolipids^4^ possibly related to the enhanced turnover of membrane lipids and altered lipoprotein metabolism^2^. Furthermore, in CF mice^2^ and then in serum from patients with CF, increased levels of saturated fatty acids (SFA) and monounsaturated fatty acids (MUFA) in combination with decreased levels of omega-3 and omega-6 polyunsaturated fatty acids (PUFA) in comparison with healthy individuals were observed^5^.

A boosted cholesterol absorption originating from impaired intestinal malabsorption and the lack of pancreatic cholesterol esterase was also observed in CF^6^ and was related to peripheral and systemic inflammation^7^, as like as the altered expression of ceramides at pulmonary levels enhances pulmonary inflammation^8^, and the modulation of ceramidase expression in CF mice seems to be a potential treatment of pulmonary inflammation^9^.

The high structural heterogeneity of lipids reflects a wide range of physicochemical properties and the involvement in a plethora of biological processes that include the regulation of the energy metabolism and storing, the structural maintenance of membranes and membrane trafficking^10,11^, the control of mucus production ^12^ as well as many signaling pathways, the calcium homeostasis, the proliferation and survival of cells controlling the aging and apoptosis machinery^13^, and the relocation of immune system cells^14^.

The great advantage of analyzing biofluids relies on the possibility to obtain biomarkers for the early-stage diagnosis and monitoring of the disease. Among the biological fluids used in the biomedical research, saliva is assumed as a plasma ultrafiltrate that mirrors the quantitative changes that take place in the blood^15,16^. Finally, the great success in considering salivary biofluid as source of disease biomarkers is due to its non-invasive and painless sampling ease, thus resulting highly tolerated and accepted also among pediatric patients if compared to blood sampling. We have previously demonstrated a relationship between salivary cytokines and the sinonasal infections severity in patients with CF^17^ and the alterations in of the salivary unsaturated and saturated non esterified fatty acids ratio in CF patients with different severity of the pulmonary phenotype^18^.

As the development of targeted therapies and medication based on phenotypic profiles is a promising alternative for CF treatment, the current study aims to compare the salivary lipidomic profile of CF patients to healthy controls (CTRL). Furthermore, considered the relevant heterogeneity of CF clinical expression^19^ we searched for specific salivary lipid signatures that may help to identify patients with different chronic obstructive pulmonary disease, *Pseudomonas Aeruginosa* (PA) infection, pancreatic insufficiency, liver disfunction and diabetes-related complications.

## Results

### CF lipidomic signature

A targeted lipidomic analysis was performed on saliva samples from 70 patients with CF and 64 CTRL. Demographic and anthropometric parameters are listed in Table 1. Genotype and clinical data are reported in Supplementary Table 1S. Globally, seven classes of lipids as: (i) ceramides (n = 70 molecules), including ceramides (Cer), hexosylceramides (HexCer), hydroxyCeramides (HydCer), (ii) diacylglycerols (DG) (n = 44), (iii) triacylglycerols (TG) (n = 242), (iv) glycerophospholipids (n = 90) including lysophosphatidylcholine (LPC), acyl-acyl phosphatidylcholine (PC aa) and acyl-alkyl phosphatidylcholine (PC ae), (v) cholesterol esters (CE) (n = 22), (vi) sphingomyelins (SM) (n = 15 molecules) and (vii) acylcarnitines (AC) (n = 40) were correctly identified and quantified. The list of lipids including names, abbreviations, and group classification is shown in Supplementary Table 2S; the lipid concentrations in each patient and CTRL are summarized in Supplementary Table 3S.

**Table 1:** Main features of CF patients and controls.

Parameters	CF patients (n = 70)	Controls (n = 64)	P value
Age (years)	30 (22–39)	31 (28–37)	0.23
Males, n (%)	37 (53)	30 (43)	0.49
Ethnicity	Caucasian	Caucasian	–
Height (cm)	169 (160–174)	162 (160–169)	0.36
Weight (kg)	65 (55–76)	60 (59–80)	0.87
BMI (kg/m^2^)	22.5 (21.1–25.6)	23.5 (22.7–24.7)	0.15
	**CF_PA** **(n = 38)**	**CF_notPA** **(n = 32)**	
Age (years)	27(22–32,75)	32,5(23–41,5)	0.15
Males, n (%)	19 (50)	14 (44)	0.41
	**CF_INS** **(n = 42)**	**CF_SUF** **(n = 28)**	
Age (years)	26 (21–32)	36 (27–44)	0.01
Males, n (%)	17 (40)	20 (71)	0.002
	**CF_LD** **(n = 12)**	**CF_notLD** **(n = 58)**	
Age (years)	30(22–40)	23(21–29)	0.22
Males, n (%)	8 (67)	29 (50)	0.19
	**CF_RD** **(n = 16)**	**CF_notRD** **(n = 54)**	
Age (years)	30(22–39)	27(22–39)	0.86
Males, n (%)	8 (50)	29 (54)	0.79

Continuous data are reported as median (interquartile range).

**Figure 1:**
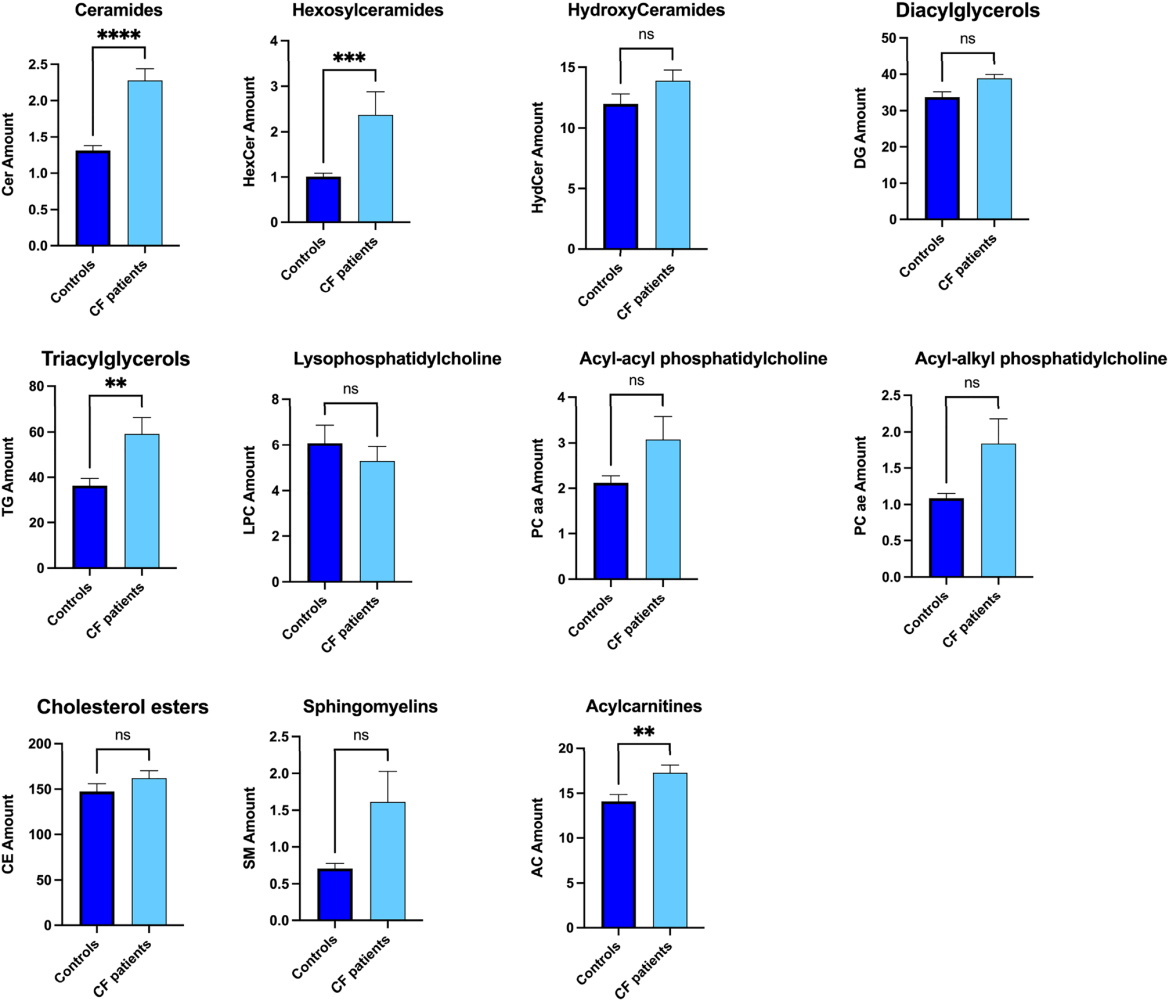
Comparison of salivary lipid levels between patients with CF (CF, n = 70) and healthy controls (CTRL, n = 64). Plots represents the lipid amount (means ± SEM), expressed as the sum of all lipid concentration within each lipid class. The significant difference was evaluated performing two-tailed unpaired parametric t-test with Welch correction in normally distributed datasets and Mann Whitney test in not-normally distributed datasets. The normal distribution was verified according to D'Agostino and Pearson test (*p < 0.05, **p < 0.01, ***p < 0.001, ****p < 0.0001, *ns* not significant).

The distribution profile of salivary lipid classes was achieved by summing all the normalized quantities of identified lipid within the single class. In saliva from patients with CF we found a significant increase of Cer, HexCer, TG and AC in respect to controls. No significant differences between the two groups were found for the salivary levels of HydCer, DG, LPC, PC aa, PC ae, CE and SM (Fig. 1).

**Figure 2:**
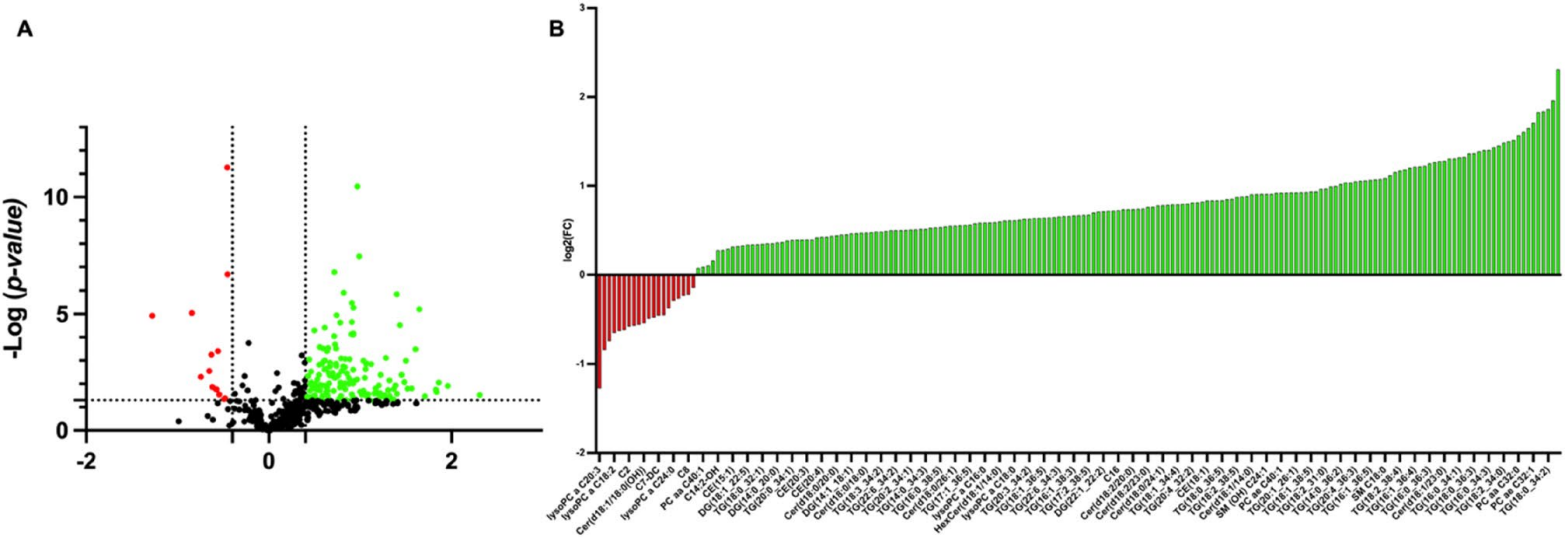
Volcano plot analysis of lipids significantly different in CF patients. (**A**) The relative abundance of each lipid was plotted against its statistical significance, respectively reported as log FC (log2 fold change) and − log10 (p-value). Red and green dots indicate features that presented both a FC > 1.5 and p-value < 0.05. Black dots refer to all the lipids identified in the dataset whose relative abundance are not significantly different between patients with CF and healthy controls. (**B**) Red and green bars refer to significantly decreased and increased lipids, respectively.

**Figure 3:**
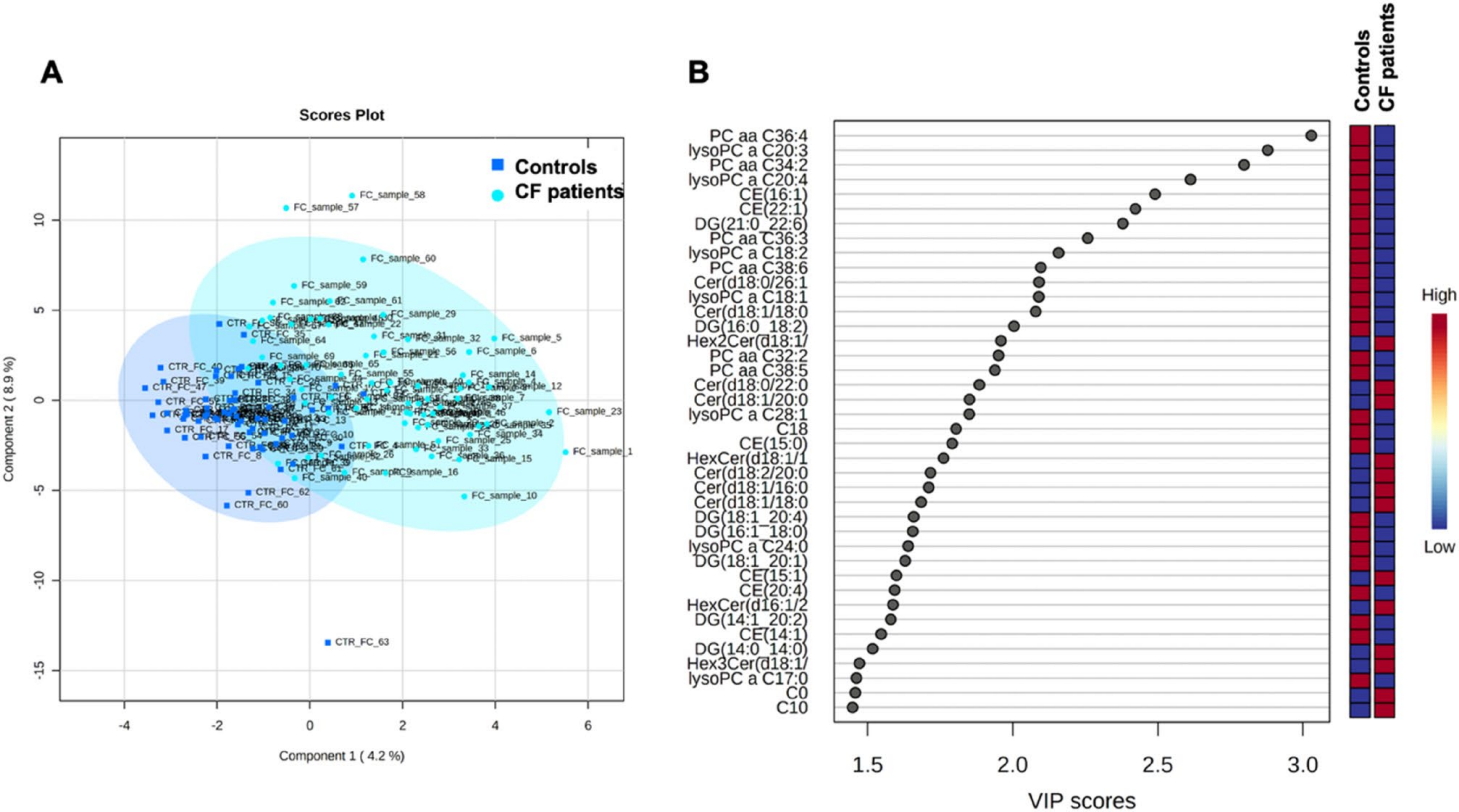
Descriptive discriminant analysis of the salivary lipidome of patients with CF. (**A**) The supervised partial least squares-discriminant analysis (PLS-DA) shows the binary segregation in CF and controls according to component 1 4.2% and component 2 8.9%. (**B**) The 36 most discriminant features identified according to the Variable Importance in Projection score (VIP > 1.5). The VIP score are assigned according to component 1. The intensity of the colored boxes represents the relative abundance in each group. The concentrations of the identified lipids were normalized, log(10) transformed, and Pareto scaled.

Univariate and multivariate statistical approaches were employed for selecting the most significant changes in lipid levels between patients with CF and CTRL. The binary comparison was performed according to volcano plot and all the lipids showing significant differences between the two groups are shown in Fig. 2. Volcano plot analysis reveals 20/195 (10,2%) lipids significantly reduced and 175/195 (89,7%) lipids significantly increased in saliva from patients with CF in comparison with CTRL, being 195 the number of significantly different lipids. To define a CF lipid signature the datasets were processed according to a supervised partial least squares-discriminant analysis (PLS-DA). Results showed lipidome clustering in two groups according to variance of the component 1 = 4.2% and component 2 = 8.9% (Fig. 3A). The Variable Importance in Projection (VIP) measure was used to identify the most discriminant lipids between CF and CTRL (Fig. 3B). In particular, the levels of PC aa C36:4, LysoPC a C20:3, PC aa C34:2, LysoPC a C20:4, CE (16:1) are able to discriminate CF patients versus CTRL.

**Figure 4:**
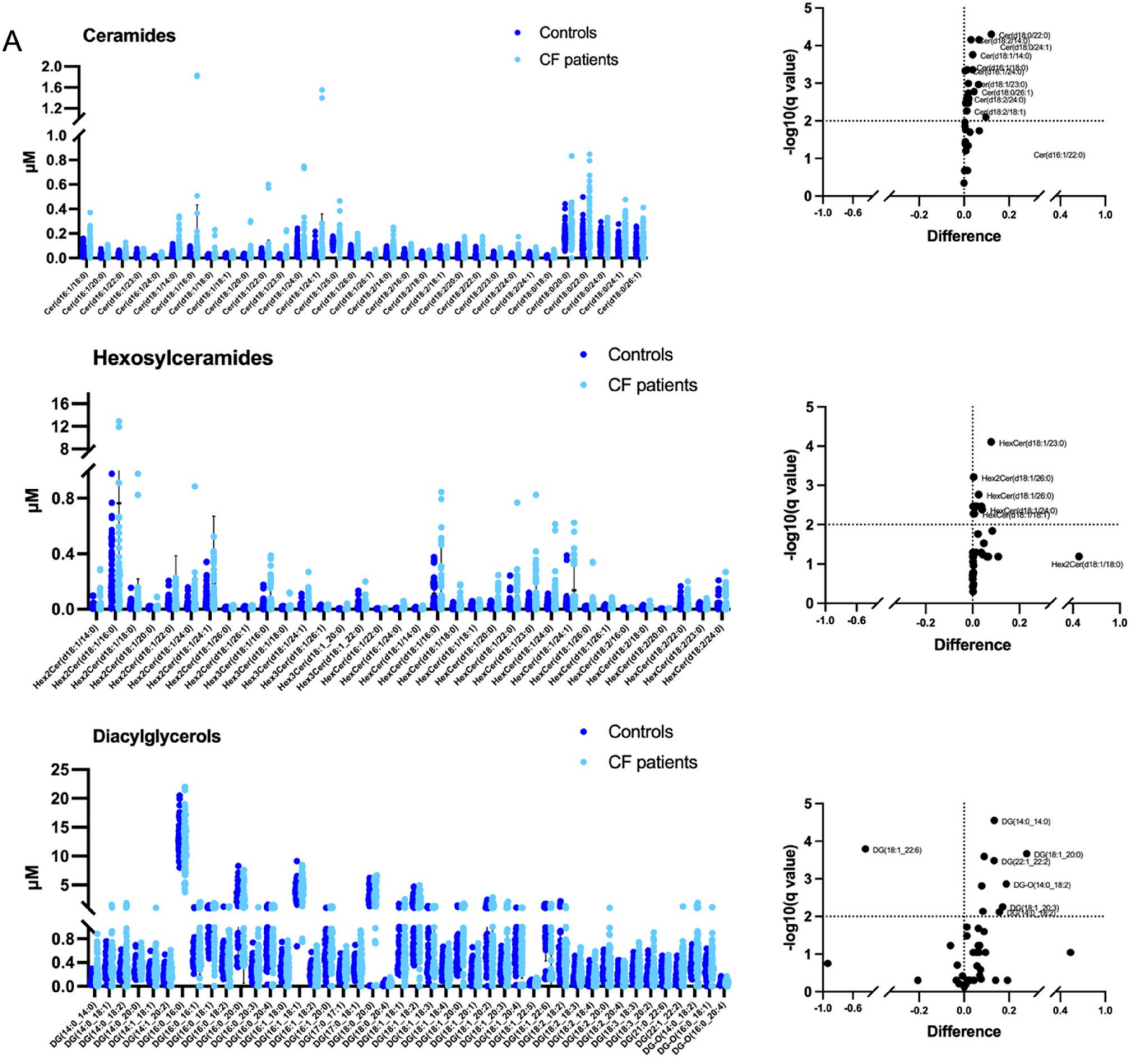

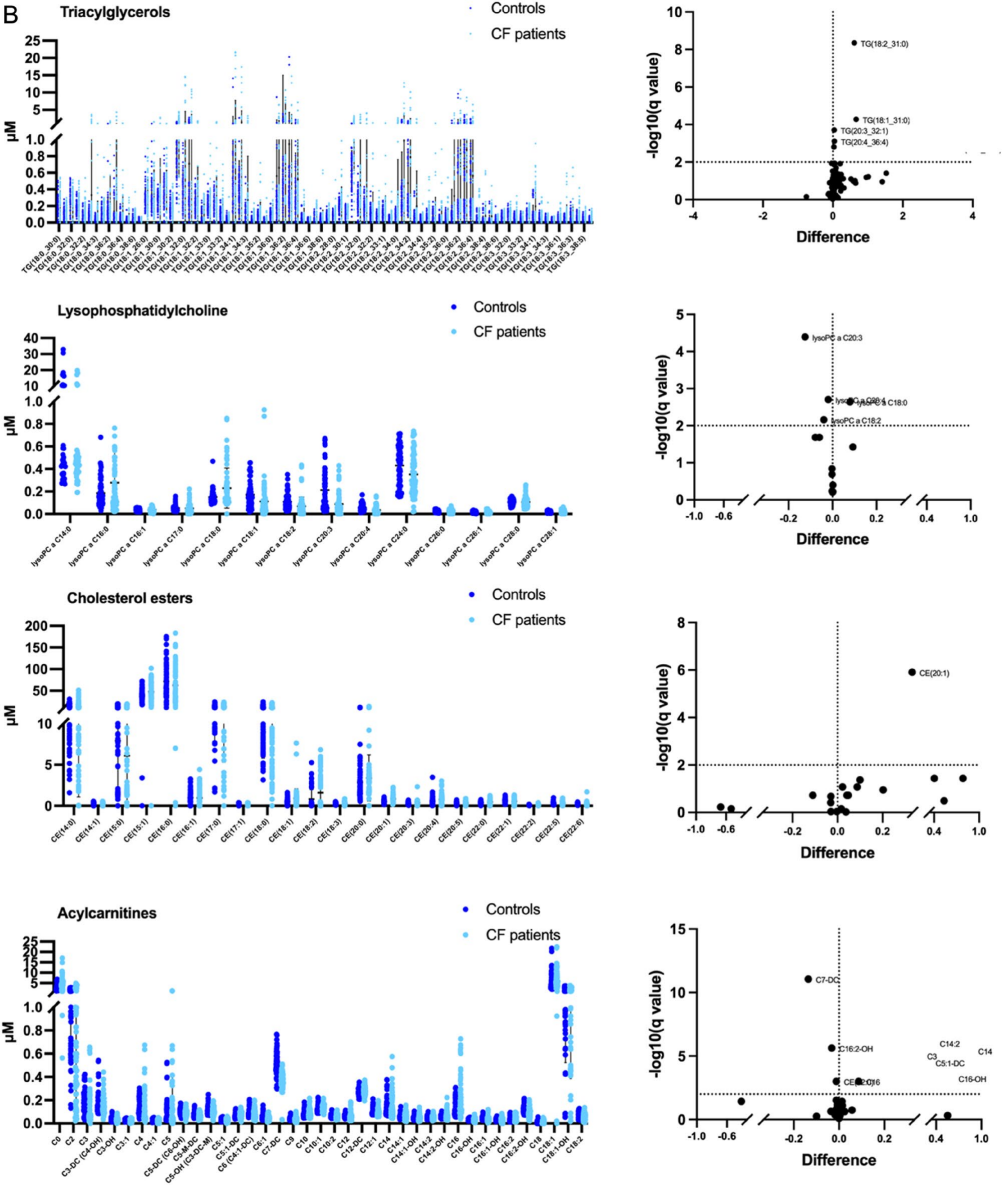
Distribution of lipid levels in saliva from CF patients. Scatter plots and volcano plots are reported to represent the salivary abundance of: (**A**) ceramides, hexosylceramides, diacylglycerols; (**B**) triacylglycerols lysophosphatidylcholine, cholesterol esters and acylcarnitines. The significantly different lipids are highlighted in volcano plots. Plots represents the lipid concentrations (means ± SD). The significant difference was evaluated performing two-tailed unpaired parametric t-test with Welch correction in normally distributed datasets and Mann Whitney test in not-normally distributed datasets. Two-stage linear step-up procedure of Benjamini, Krieger and Yekutieli was performed as False Discovery Rate approach (Q = 1%). The normal distribution was verified according to D'Agostino and Pearson test.

The binary comparison within each subclass of lipids was performed in order to highlight molecules more related to CF (Fig. 4). The subclasses with the highest number of significantly different lipids in saliva from patients with CF included Cer, HexCer and DG (Fig. 4A). Differences in salivary levels were observed also for TG, LPC, CE and AC subclasses (Fig. 4B).

In details, the percentage of significantly different lipids was 53%, 35%, 23%, 2.5%, 28%, 4.5% and 10% within Cer, HexCer, DG, TG, LPC, CE and AC subclasses, respectively.

The list of lipids significantly different in saliva from CF patients versus CTRL was summarized in Table 2. All lipids determined to be significantly different by p-value were also significant by q-value.

**Table 2:** Differentially abundant lipids (microM) in the CF saliva versus controls.

Lipid subclass	q-value	p-value	Mean of controls	Mean of CF patients	Difference	ratio
**Ceramides**						
Cer(d16:1/18:0)	0.000439	0.000163	0.0668	0.1047	0.0379	1.5671
Cer(d16:1/20:0)	0.000439	0.000163	0.0204	0.0341	0.0137	1.6683
Cer(d16:1/24:0)	0.000467	0.000202	0.0095	0.0144	0.0048	1.5084
Cer(d18:0/20:0)	0.001088	0.000606	0.1787	0.2429	0.0642	1.3593
Cer(d18:0/22:0)	0.00005	0.000003	0.1380	0.2593	0.1214	1.8790
Cer(d18:0/24:1)	0.00007	0.000013	0.0916	0.1575	0.0658	1.7189
Cer(d18:0/26:1)	0.001672	0.001035	0.0951	0.1393	0.0442	1.4646
Cer(d18:1/14:0)	0.000175	0.000043	0.0436	0.0816	0.0380	1.8714
Cer(d18:1/16:0)	0.00795	0.009347	0.0336	0.1307	0.0971	3.8934
Cer(d18:1/18:0)	0.005448	0.006026	0.0166	0.0285	0.0120	1.7218
Cer(d18:1/20:0)	0.003358	0.003325	0.0138	0,0321	0,0184	2.3367
Cer(d18:1/23:0)	0.001014	0.000502	0.0128	0.0310	0.0183	2.4288
Cer(d18:1/26:0)	0.002565	0.00195	0.0400	0.0611	0.0210	1.5254
Cer(d18:2/14:0)	0.00007	0.000012	0.0354	0.0665	0.0311	1.8778
Cer(d18:2/16:0)	0.003033	0.002627	0.0227	0.0331	0.0105	1.4627
Cer(d18:2/18:1)	0.005448	0.006069	0.0259	0.0374	0.0115	1.4462
Cer(d18:2/20:0)	0.001821	0.001239	0.0286	0.0477	0.0191	1.6698
Cer(d18:2/23:0)	0.003358	0.003202	0.0109	0.0184	0.0076	1.6952
Cer(d18:2/24:0)	0.002565	0.002063	0.0158	0.0274	0.0117	1.7405
**Hexosylceramides**						
Hex2Cer(d18:1/26:0)	0.000617	0.000056	0.0053	0.0101	0.0048	1.8977
Hex3Cer(d18:1/16:0)	0.003421	0.001018	0.0361	0.0740	0.0379	2.0513
Hex3Cer(d18:1/24:1)	0.003421	0.001382	0.0263	0.0462	0.0199	1.7585
HexCer(d16:1/24:0)	0.005278	0.002794	0.0044	0.0080	0.0036	1.8063
HexCer(d18:1/14:0)	0.003421	0.000833	0.0053	0.0080	0.0027	1.5145
HexCer(d18:1/18:0)	0.003421	0.001	0.0124	0.0269	0.0145	2.1738
HexCer(d18:1/18:1)	0.005278	0.00285	0.0234	0.0314	0.0081	1.3452
HexCer(d18:1/20:0)	0.003421	0.001346	0.0247	0.0441	0.0194	1.7851
HexCer(d18:1/23:0)	0.000078	0.000004	0.0373	0.1169	0.0796	3.1340
HexCer(d18:1/24:0)	0.004155	0.00187	0.0378	0.0789	0.0411	2.0860
HexCer(d18:1/26:0)	0.001718	0.000232	0.0126	0.0384	0.0258	3.0469
HexCer(d18:1/26:1)	0.003421	0.001386	0.0124	0.0197	0.0073	1.5926
**Diacylglycerols**						
DG-O(14:0_18:2)	0.001366	0.000239	0.3378	0.5254	0.1876	1.5554
DG(14:0_14:0)	0.000028	< 0.000001	0.0817	0.2158	0.1341	2.6410
DG(14:0_18:2)	0.007583	0.002208	0.2519	0.4087	0.1569	1.6225
DG(14:0_20:0)	0.001552	0.000316	0.2744	0.3530	0.0785	1.2864
DG(14:1_18:1)	0.007303	0.001914	0.2203	0,3038	0.0835	1.3790
DG(17:0_17:1)	0.000255	0.00003	0.2146	0.3034	0.0888	1.4138
DG(18:1_20:0)	0.000215	0.000019	0.5280	0.8065	0.2785	1.5275
DG(18:1_20:3)	0.005596	0.001304	0.3140	0.4858	0.1718	1.5471
DG(18:1_22:6)	0.000161	0.000009	0.9782	0.5458	− 0.4325	0.5580
DG(22:1_22:2)	0.00033	0.000048	0.2075	0.3406	0.1331	1.6414
**Triacylglycerols**						
TG(14:0_38:5)	0.001562	0.000039	0.0310	0.0590	0.0280	1.9020
TG(18:1_31:0)	0.000052	< 0.000001	0.8639	1.5270	0.6633	1.7676
TG(18:2_31:0)	< 0.000001	< 0.000001	0.6381	1.2510	0.6128	1.9605
TG(20:1_31:0)	0.000002	< 0.000001	3.4890	6.9370	3.4480	1.9882
TG(20:3_32:1)	0.000192	0.000003	0.0434	0.0826	0.0392	1.9017
TG(20:4_36:4)	0.000769	0.000016	0.0251	0.0678	0.0427	2.7008
**Cholesterol esters**						
CE(20:1)	0.000001	< 0.000001	0.5129	0.8441	0.3312	1.6457
**Lysophosphatidylcholine**						
lysoPC a C18:0	0.002285	0.000679	0.1503	0.2297	0,0795	1.5283
lysoPC a C18:2	0.006928	0.002744	0.1088	0.0693	− 0.0395	0.6367
lysoPC a C20:3	0.000041	0.000004	0.2124	0.0877	− 0.1247	0.4131
lysoPC a C20:4	0.001973	0.000391	0.0542	0.0350	− 0.0191	0.6466
**Phosphatidylcholine**						
PC ae C44:5	0.008677	0.000232	0.0265	0.0389	0.0125	1.4718
**Acylcarnitines**						
C0	0.000138	0.000012	2.8400	4.7560	1.9160	1.6746
C16	0.001126	0.000127	0.1323	0.2188	0.0865	1.6538
C16:2-OH	0.000002	< 0.000001	0.1215	0.0890	− 0.0326	0.7322
C7-DC	< 0.000001	< 0.000001	0.5043	0.3682	− 0.1361	0.7301

**Figure 5:**
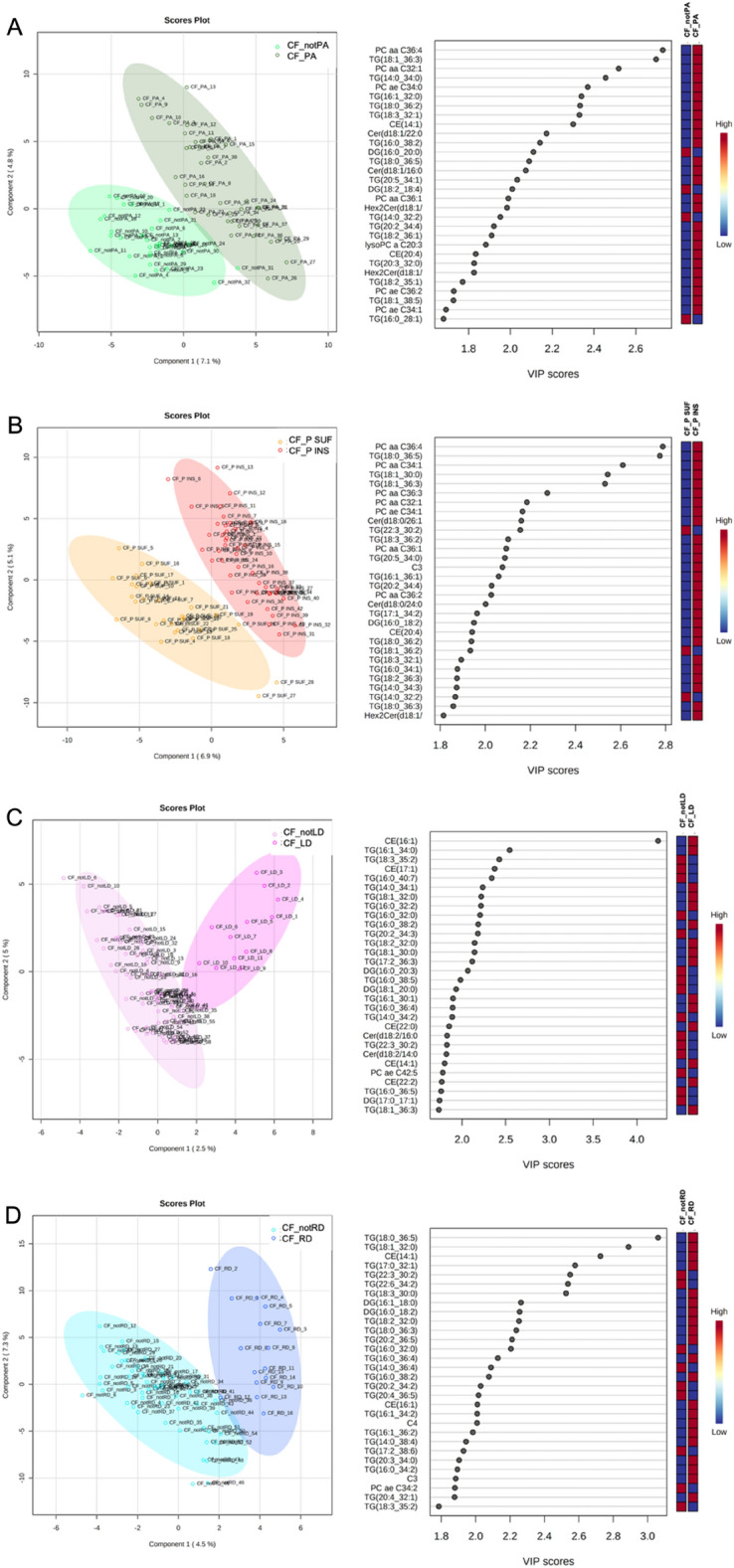
The supervised partial least squares-discriminant analysis (PLS-DA) plots, show the lipid segregation between patients with and without colonization by *Pseudomonas Aeruginosa* (**A**); patients with pancreatic sufficiency and insufficiency (**B**); patients with and without liver disfunction (**C**) and patients with or without diabetes related complication (**D**). The VIP score are assigned according to component 1. The 30 most important lipids, according to their VIP score are reported. The intensity of the colored boxes denotes the relative lipid abundance in each group of patients.

To evaluate CF clinical phenotype on CF lipidome, the sample cohort employed for this study was subdivided in patients belonging to distinct CF classes according to diagnosis of (i) PA infection (n = 16 CF_notPA and n = 19 CF_PA subgroups), (ii) pancreatic insufficiency (n = 30 CF_SUF and n = 40 CF_INS), (iii) related liver disease (n = 12 CF_LD and n = 58 CF_RD) and related diabetes (n = 16 CF_RD and n = 54 CF_notRD). The four datasets of lipid concentrations were transformed according to log(10) and Pareto scaling (Supplementary Fig. 1S). Binary comparisons between CF_notPA and CF_PA subgroups (Fig. 5A) resulted in the identification of discriminant lipids according to component 1 (VIP > 2.5), PCaa C36:4, TG(18:1_36:3), TG(14:0_34:0) and PCaa C32:1. The significantly different lipid concentrations in CTR_CF, CF_notPA, and CF_PA were evaluated performing the ANOVA analysis (Supplementary Fig. 2).

Binary comparisons between CF_SUF and CF_INS subgroups (Fig. 5B), Supplementary Fig. 3) resulted in the identification of discriminant lipids according to component 1 (VIP > 2.5), PCaa C36:4, TG(18:0_36:5), PCaa C34:1, TG(18:1_36:3). The significantly different lipid concentrations in CTR_CF, CF_P SUF, and CF_P INS were evaluated performing the ANOVA analysis (Supplementary Fig. 3). Unfortunately, the contribute of pancreatic insufficiency in present CF cohort could not deeply evaluated because the observed differences are not independent on demographic parameters (Table 1).

**Figure 6:**
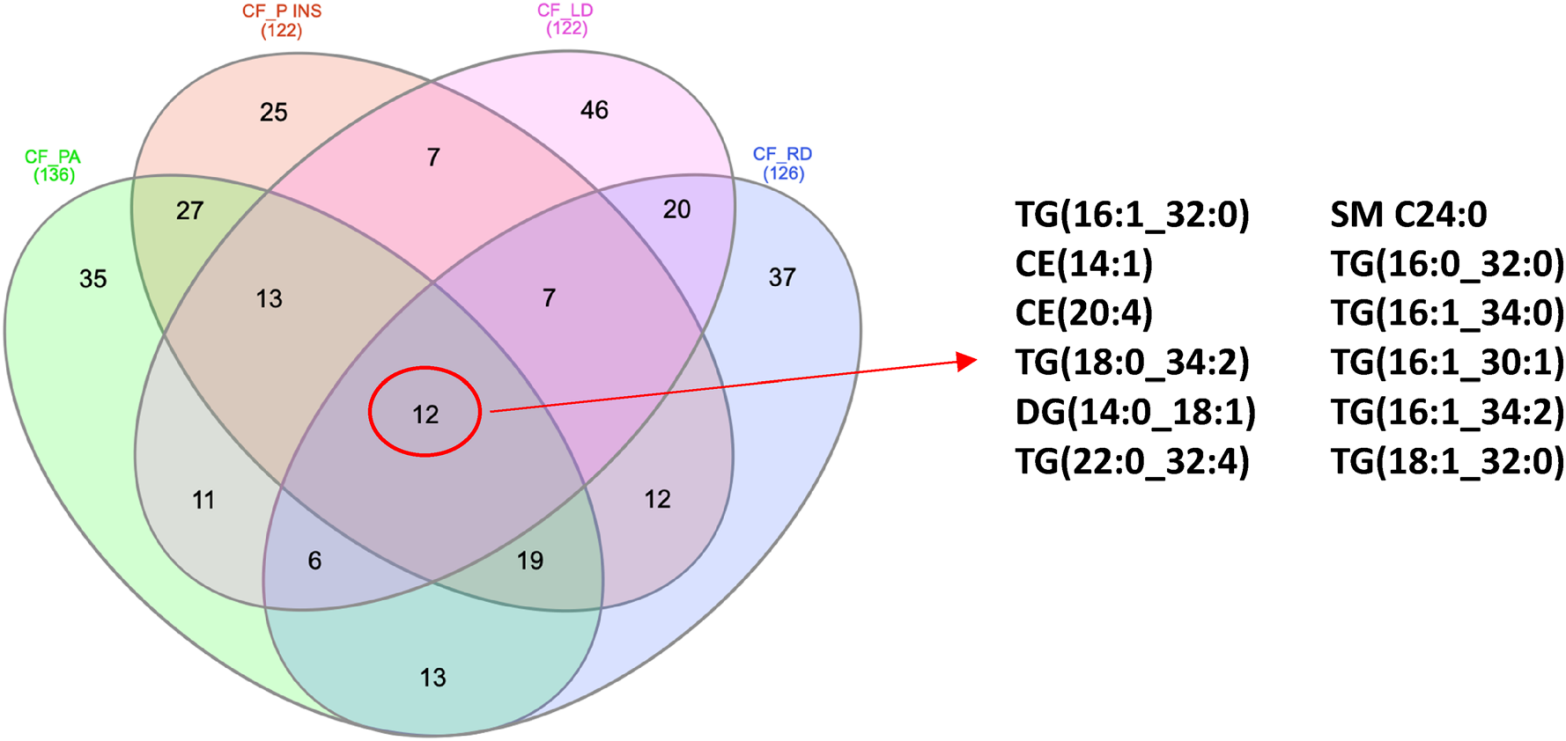
Eulero-Venn diagram reports the common lipids characterizing by a discriminat VIP score (VIP > 1) in four phenotypic groups CF_PA, CF_P INS, CF_LD and CF_RD groups. The 12 common molecules are showed.

**Figure 7:**
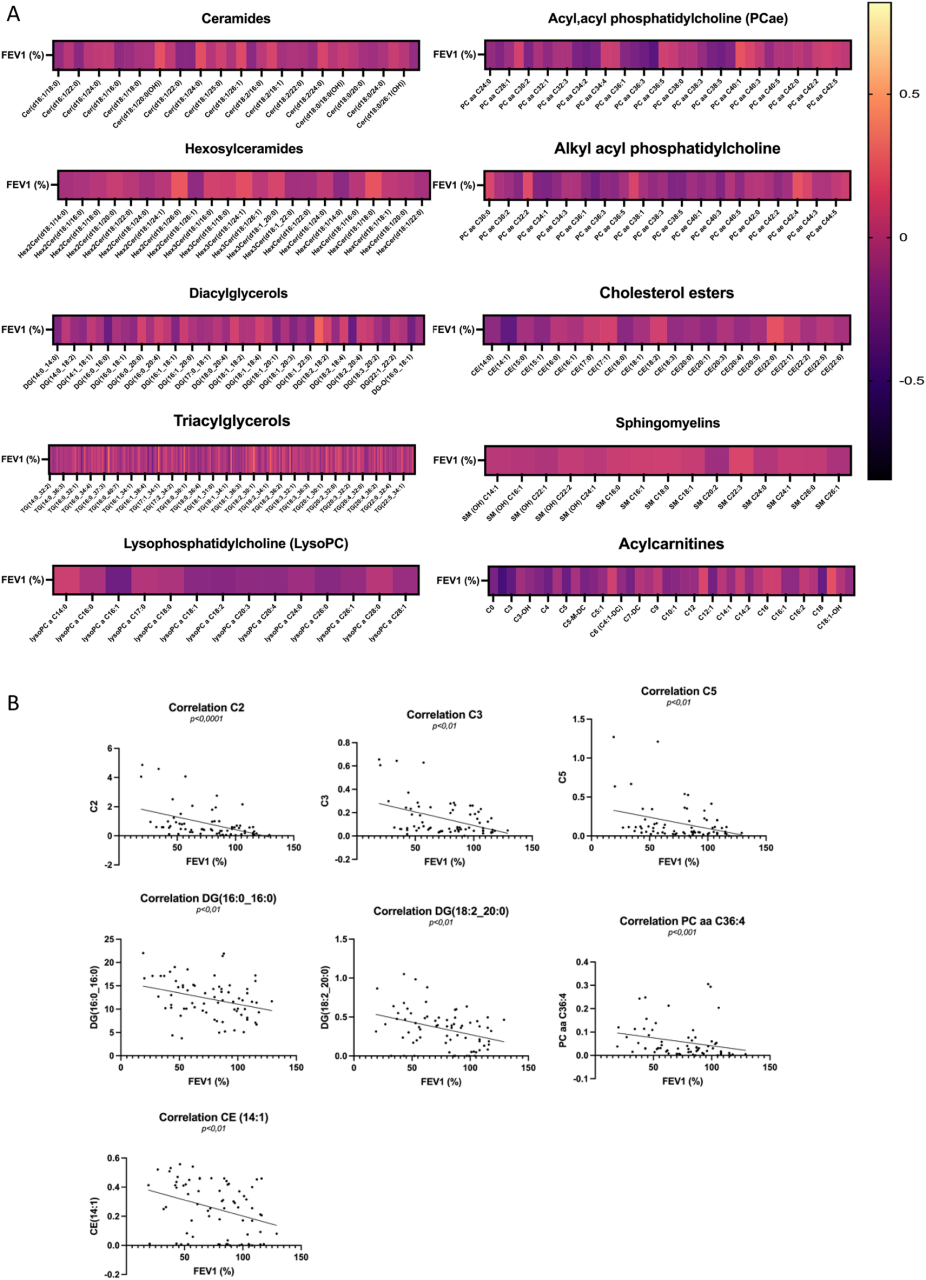
Spearman’s rank correlations between FEV1% and lipids in CF patients are reported. (**A**) All molecule concentrations belong to different lipid classes were correlates with FEV1% parameter of CF patients. The negative and positive association was represented by dark black and light yellow, respectively. (**B**) Significant associations between FEV1% and C2 (p < 0.0001), C3 (p < 0.01), C5 (p < 0.01), DG(16:0_16:0) (p < 0.01), DG(18:2_20:0) (p < 0.01), PC aa C36:4 (p < 0.001) and CE(14:1) (p < 0.01) are reported.

Binary comparisons between CF_notLD and CF_LD subgroups (Fig. 5C) resulted in the identification of discriminant lipids according to component 1 (VIP > 2.5), CE(16:1), TG(18:3_35:2). The significantly different lipid concentrations in CTR_CF, CF_notLD, and CF_LD were evaluated performing the ANOVA analysis (Supplementary Fig. 4). Binary comparisons between CF_notRD and CF_RD subgroups (Fig. 5D) resulted in the identification of discriminant lipids according to component 1 (VIP > 2.5), TG(18:0_36:5), TG(18:1_32:0), CE(14:1), TG(17:0_32:1), TG(22:3_30:2), TG(22:6_34:2). The specific signature involve a set of twelve altered lipids common to all binary comparisons, including TG(16:1_32:0), CE(14:1),CE(20:4), TG(18:0_34:2), DG(14:0_18:1), TG(22:0_32:4), SM C24:0, TG(16:0_32:0), TG(16:1_34:0), TG(16:1_30:1), TG(16:1_34:2), TG(18:1_32:0), as reported in Fig. 6. Finally, the relationship between FEV1% values and lipidome was investigated. A correlation matrix based on Spearman correlation distance was carried out to explore the correlations among FEV1% parameter and all detected lipids in CF patients within each specific class (Fig. 7A). The analysis revealed significant negative correlations between FEV1% and C2 (*p* < 0.0001), C3 (*p* < 0.01), C5 (*p* < 0.01), DG(16:0_16:0) (*p* < 0.01), DG(18:2_20:0) (*p* < 0.01), PC aa 36:4 (*p* < 0.001), CE14 (*p* < 0.01) (Fig. 7B).

## Discussion

**Figure 8:**
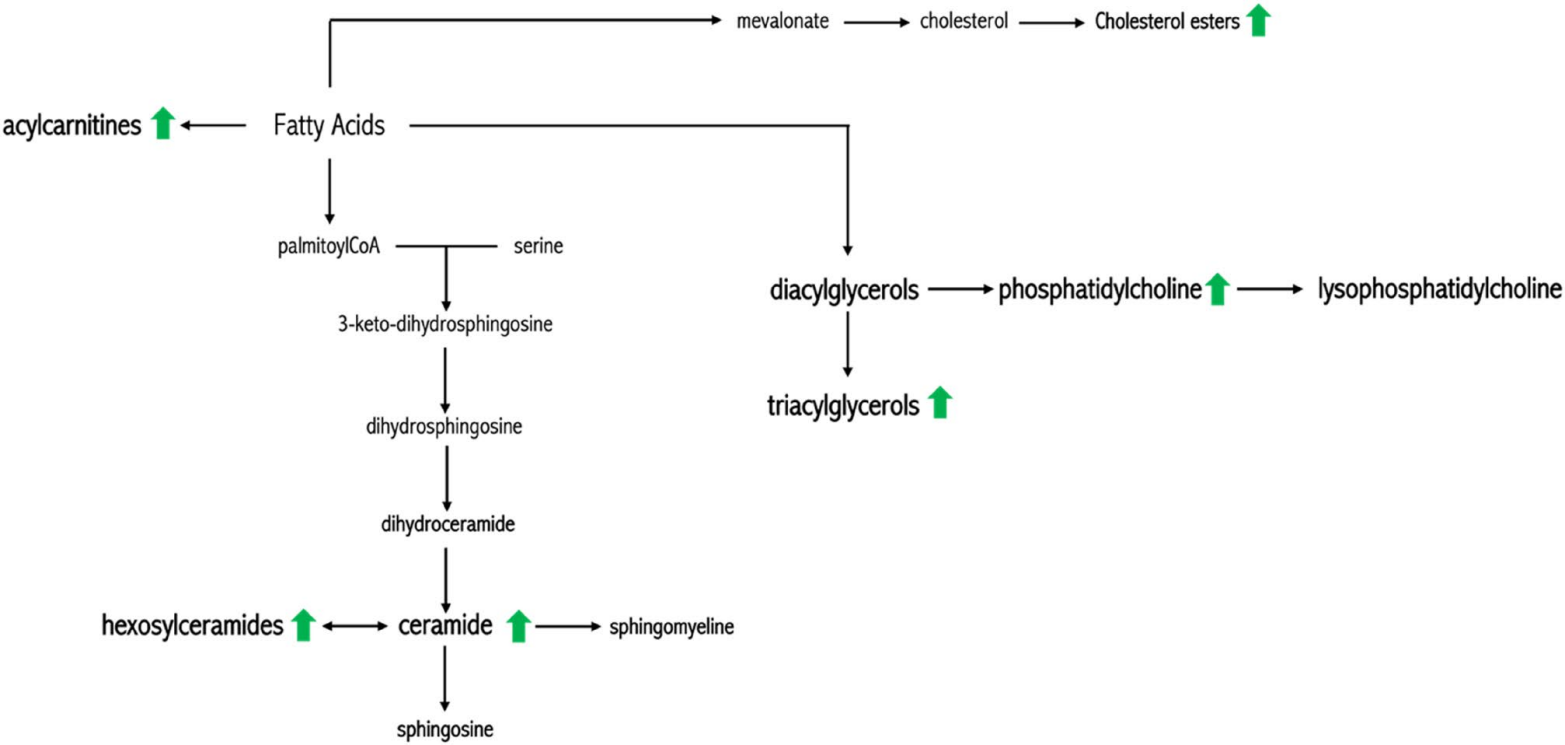
Summary of lipid biosynthetic pathways. Green arrows represented the more abundant lipid classes in CF saliva.

Targeted mass spectrometry-based lipidomic platform was used to measure lipids belonging to seven different classes, to define the salivary lipid profile of CF patients in comparison to healthy controls. Our study revealed the significant increase of specific lipid classes as ceramides and hexosylceramides, triglycerides and acylcarnitine in saliva from CF patients (Fig. 8), and specific lipid profiles associated to the disease phenotype (i.e., chronic obstructive pulmonary disease, *Pseudomonas Aeruginosa* infection, pancreatic insufficiency, liver disfunction and diabetes-related complications).

Ceramides are implicated in inflammation processes, and their increase in tissues from CF animal models^20^ and in respiratory cells^21^ tissues and biofluids like serum and bronchoalveolar fluid from patients with CF is well known^21,22^. Such accumulation stimulate the neutrophil and macrophage recruitment^8,23^ with the following induction of cell death^24^ that results in the formation of cell fragments and DNA deposits that helps bacterial adherence.

Furthermore, ceramides contribute to enhance the CF inflammatory response^25,26^ stimulating the release of IL-1 and other cytokines^27^ and through the activation of the NFkB pathway^28^. In addition, they help the colonization by *Pseudomonas Aeruginosa*^8^ and mediate the development of lung fibrosis in mice^29^. As the accumulation of ceramides depends on the cumulative alteration in recycle biosynthetic reactions mediated by sphingomyelinase and ceramidases^8^, the targeting of such enzymes has been evaluated in CF cells with a relevant improvement of CF inflammation^30^. Additional studies were replicated with encouraging results in the CF mice model^9^. These treatments may impact on patients with CF^9,21^ and the analysis of ceramides in saliva may help to identify CF patients eligible for the treatment and to easily monitor the effects.

Ceramide accumulation depends on (i) de novo anabolic synthetic pathway with the condensation of the amino acid, L-serine, and palmitoyl-CoA, producing 3-ketosphingonine or (ii) enzymatic activities of ceramidases. Five human ceramidases have been identified and classified according to their optimal pH for catalytic activity: acid ceramidase (ACDase) encoded by ASAH1 gene, neutral ceramidase (NCDase) encoded by ASAH2, and alkaline ceramidases (ALKCDase) encoded by the genes ACER1, ACER2 and ACER3^31^. According to human atlas databases (https://www.proteinatlas.org) the acid ceramidases, ASH1, is expressed in respiratory system, as nasopharynx, bronchus and lung. Specifically, the alkaline ceramidase 3 (ACER3) is highly expressed in the lung. It was demonstrated that the ASH1 inhibition is linked to Cystic Fibrosis; conversely, the ACER3 inhibition is associated to bacterial infection proliferation^32^. We speculate about a ceramide accumulation, via putative ACER3 impairment in lung of CF patients. As ceramidases the key enzymes in cell survival maintaining, their potential therapeutic role could be investigate^33^*.* Despite evidences, showing an increment of ceramides associated with the increased inflammation and grater susceptibility to infection in CF, Westholter et al.^34^ demonstrated a deficit of sphingosine and ceramides. However, the authors discuss about an imbalanced ceramides composition in blood from a large cohort of CF patients suggesting an alteration of specific ceramides subtypes rather than a general upregulation of ceramides in CF serum. In addition, the ceramides accumulation could be related to increased sphingolipid levels and sphingomyelinase activity^35^.

Furthermore, we measured a significant increase of total TG in saliva from patients with CF in agreement with various results on plasma samples^36,37^. The high levels of TG could be due to the increased rate of synthesis that compensate the reduced intestinal absorption, as we recently demonstrated for cholesterol in the CF mice model^7^ or to the decreased clearance. It may be related also to the increased insulin levels often observed in patients with CF^38^ even if future studies are required to define the relationships between TG and insulin levels. The TG accumulation enhances the production of proinflammatory cytokines, such as IL1β, IL-6, IL-8 and TNFα that are hallmarks of chronic inflammation and severe stress in CF. Furthermore, cytokines inhibit lipoprotein-lipase (thus reducing the catabolism of lipoproteins with the subsequent storing of TG in the adipocytes) and potentiate hepatic lipogenesis^39,40^ inducing a vicious circle that causes a further increase of circulating TG. In agreement, enhanced lipogenesis and delta-7 desaturase activity due to an increase in the expression of SREBP-1c was described in cellular model of CF^41^. Thus, although most studies focused on the correction of the altered nutrient status in patients with CF^42^ more attention should be payed to patients with high levels of TG in serum and/or in saliva in order to contrast the pro-inflammatory and atherogenic effects of such molecules^37^. Finally, the analysis of TG in saliva may help to select the patients that need to be treated and to monitor the therapy effects.

Despite the lower levels of AC reported in cord blood and in serum from newborn with CF^43^, we found that AC was increased in saliva from adult CF patients, in agreement with the slight increase reported in serum from adult CF patients^44^. The results are consistent with the lipogenesis activation and with the decreased activity of $\beta$-oxidation of fatty acids in CF^45^. On the other hand, mitochondrial dysfunctions in CF patients were previously described with abnormal mitochondrial morphology, higher cellular respiration and higher calcium mitochondrial uptake^45,46^. Disfunctions may enhance inflammation and oxidative stress, as a consequence of deep imbalance between oxidants and anti-oxidants species^45^. Thus, the analysis of AC in saliva from patients with CF may also represent a surrogate biomarker of mitochondrial dysfunction in CF.

Interestingly, our study identified also a lipid signature that include PC a C36:4, lysoPC a C20:3, PC aa C34:2 and lysoPC a C20:4 and was able to discriminate CF from healthy controls. The present lipid panel need to be validated on a larger cohort of patients with CF and then prospectively evaluated in the clinical context. It may help to discriminate patients with CF from cases with CFTR-related disorders^7^ or, in the context of the newborn screening for CF, to identify the cases with CF-screening positive inconclusive diagnosis with a higher risk to evolve in CF^47^.

Unfortunately, in this study, the carrier status of control subjects was not evaluated. A future direction would be to compare the lipidomic profile of CF patients versus CF carriers in order to better define the role of CFTR dysfunction in the pathogenesis of lipidomic alterations.

Finally, we observed that salivary CF lipidome may change accordingly to the severity of the disease. Our binary comparative analysis applied to different subgroups of CF patients according to their specific clinical expression demonstrated specific salivary lipid signatures associated to the main parameters of clinical severity like chronic obstructive pulmonary disease, *Pseudomonas Aeruginosa* infection, pancreatic insufficiency, liver disfunction and diabetes-related complications.

The data of our study need to be confirmed on larger populations in prospective studies; furthermore, limitations of the study are the lack of a group of carrier subjects to be compared to CF patients, and the different diet and molecular therapies (i.e., correctors and potentiator) that several patients are performing, and that may impact on the lipidomic profile. Such parameters would be considered in future studies. In any case, the present study suggests that salivary lipid signatures are related to CF and to the severity and complications of the disease.

## Limitation of study

Our results provide a comparison between lipidome characterizing CF patients cohort in respect to healthy controls. Unfortunately, it was based on a cohort of controls that was not screened to determine the percentage of CF carriers. The carrier status of the controls was not evaluated because the study was conducted using a small cohort of healthy controls. As the incidence of CF carriers is defined as 1:25, only 2 subjects among the 64 controls in this cohort of healthy subjects could be CF carriers. Future study will be needed for extending the understanding of lipid dysregulation in CF patients comparing CF to non-CF carrier as well as CF carrier to. It will be powerful highlighting the argument that the lipid imbalances are due to CFTR dysfunction and not CF interventions. The size of cohort influenced also the study concerning the affection of pancreatic insufficiency. Indeed, the contribute of pancreatic insufficiency in present CF cohort could not evaluated because the observed differences are not independent on demographic parameters.

Moreover, the CF patients in cohort exhibit different therapeutics plans based on the introduction of small molecules as modulators and/or integrators in their diet. As the patients started therapies in different periods, it is not possible to evaluate the confounder effect correctly.

In addition, it was not possible to investigate how insulin levels affect TG levels in the saliva due to the not availability of serum insulin dosages of patients. Finally, the use of lipids to differentiate the subgroups of CF represents a pilot study and further research is necessary.

## Methods

### Study population

We recruited 70 adults with CF with a median age of 30 years (range: 18–62 years; 53% males) and 64 healthy volunteers with a median age of 32 years (range: 22–66 years; 33% males) as control (CTRL) group. The study was approved by the Ethics Committee of Federico II University Hospital and was performed according to the current version of the Helsinki Declaration. Written informed consent was obtained from all patients and healthy volunteers enrolled. The diagnosis of CF was performed according to current guidelines^48^. The *CFTR* genotype was defined through the screening of the most frequent mutations and rearrangements and gene sequencing was performed when mutations were not detected in one or both alleles^49,50^. All 70 patients were homozygous or compound heterozygous for severe (i.e., class I-III) *CFTR* mutations.

### Sample collection

Whole resting saliva samples (obtained without mechanical or chemical stimulation) were collected between 9 a.m. and 12 a.m., as previously described^47^. The patients were instructed not to drink or eat anything except water for 2 h before the sample collection. One to three milliliters of whole resting saliva were collected in sterile plastic tubes, which were chilled on ice during saliva collection. Immediately after collection, the samples were centrifuged for 30 min at 14,000×*g* to remove bacteria/cellular debris and the supernatants were stored at 80 °C.

### Clinical evaluation

The most recent forced expiratory volume in 1 s (FEV1) of CF patients was evaluated while they were clinically stable within the previous year^19^. The FEV1 was expressed as the percentage of predicted value for age, according to standardized reference equations for spirometry^51^. The median FEV1 in CF patients resulted 82% (range: 19–129%) and on the basis of such equation the patients with CF could be classified as severe or mild pulmonary disease^19^. Chronic obstructive pulmonary disease was evaluated according to FEV1% values, listed in Supplementary Table 1S.

Airway colonization by PA was identified by sputum or oropharyngeal swab culture^52^. The colonization was observed in patients with CF. Pancreatic insufficiency was defined on the basis of fecal pancreatic elastase lower than 200 µg/g feces measured in the absence of acute pancreatitis or gastrointestinal diseases^53^. Liver disease was evaluated by means of clinical, biochemical or ultrasonography data recorded in two consecutive examinations within a 3-month period, in the absence of other known causes of chronic liver disease^19^. Patients were considered as affected by CFLD when they had liver cirrhosis, with imaging techniques showing nodular hepatic parenchyma and signs of portal hypertension^19^. The diagnosis of CFRD was made according to the standard American Diabetes Association criteria^19^. To performe cut-off analysis evaluating the *Pseudomonas Aeruginosa* infection, pancreatic insufficiency, liver disfunction and diabetes-related the 70 CF patients were subdivided as follow: 38 patients were affected by PA infection (n = 38 CF_PA and n = 32 CF_notPA subgroups), 42 patients were affected by pancreatic insufficiency (n = 42 CF_INS and n = 28 CF_SUF, 12 patients had CF-related liver disease (n = 12 CF_LD and n = 58 CF_notLD) and 16 patients had CFTR-related diabetes (n = 16 CF_RD and n = 54 CF_notRD) (Table 1 and Supplementary Table 1S).

### LC-MSMS targeted methods

Aliquots of 100 µL of saliva from CF patients and healthy volunteers were dissolved in 300 μL isopropanol. The solution was vortexed and centrifuged at 4000 rpm (5424R Centrifuge, FA-45-24-11 Rotor, Eppendorf) at 10 °C for 15 min. An aliquot of 50 μL of the saliva mixture was subsequently analyzed described below. Saliva lipidome content was characterized by using targeted metabolomics^54,55^. According to Mxp Quant 500 protocols (Biocrates Life Sciences Innsbruck, Austria), the lipidomic mass spectrometry-based platform was set to measure the concentration of 523 lipids including (i) ceramides (n = 70 molecules), including ceramides (Cer), hexosylceramides (HexCer), hydroxyCeramides (HydCer), (ii) diacylglycerols (DG) (n = 44), (iii) triacylglycerols (TG) (n = 242), (iv) glycerophospholipids (n = 90) including lysophosphatidylcholine (LPC), acyl-acyl phosphatidylcholine (PC aa) and acyl-alkyl phosphatidylcholine (PC ae), (v) cholesterol esters (CE) (n = 22), (vi) sphingomyelins (SM) (n = 15 molecules) and (vii) acylcarnitines (AC) (n = 40). Three technical replicates were carried out for each sample. Aliquots of saliva mixture, containing 50 μg of proteins, were transferred onto a 96-well extraction plate containing the positions for blanks, PBS, calibrants, and quality controls (QC), and dried under nitrogen stream. The proteins concentration of saliva mixture was in [0.8–1.30] μg/μL; saliva volumes in [38.5–62.5] μL were loaded. Then, each sample was incubated for 1 h with 50 μL of 5% phenyl isothiocyanate (PITC) solution to derivatize amino acids and biogenic amines and dried again^56^. Lipids were extracted with 300 μL of 5 mM ammonium acetate in methanol in the shaker during 30 min at 450 rpm and eluted in a new 96-well plate by centrifugation. Aliquots of 10 μL of each extract were diluted for tandem mass spectrometry analysis^57,58^.The mass spectrometry analysis was performed setting the multiple reaction monitoring (MRM) in order to identify and quantify unique molecular species belonging to different lipid classes. Direct flow injection analysis (FIA) was carried out using Agilent 1260 Infinity II HPLC (High-performance liquid chromatography), online with a Triple Quad 5500 + System QTrap-Ready (AB Sciex). The injection volume into the system was 20 μL within the mobile phase (FIA solvent) at an initial flow rate of 0.03 mL/min until 1.6 min, followed by flow rates of 0.20 mL/min for 1.6 min and 0.02 mL/min for 0.20 min. The auto sampler was cooled at 10 °C. The ESI (Electrospray ionization) source operated in positive ion mode using the following parameters: spray voltage 55 kV, temperature of 450 °C, GS1 (Ion Source Gas 1) 20, GS2 (Ion Source Gas 1) 40, CUR (Curtain Gas) 30, CAD (Collision Gas) 8. Data were acquired using Analyst software (version 1.7.1 Ab Sciex) and data analysis was performed used MetIDQ Oxygen 2976 (Biocrates Life Sciences Innsbruck, Austria). In details, the lipids were detected using two different acquisition methods: (i) FIA method 1 detected lysophosphatidylcholine (LPC), acyl-acyl phosphatidylcholine (PC aa), acyl-alkyl phosphatidylcholine (PC ae) and acylcarnitines (AC). The ESI source operated in positive ion mode using the following parameters: spray voltage 55 kV, temperature of 450 °C, GS1 20, GS2 40, CUR 30, CAD 8; ii) FIA method 2 detected: ceramides, diacylglycerols (DG), triacylglycerols (TG) cholesterol esters (CE), sphingomyelins (SM). The ESI source operated in positive ion mode using the following parameters: spray voltage 55 kV, temperature of 300 °C, GS1 20, GS2 50, CUR 30, CAD 6. Data were acquired using Analyst software (version 1.7.1 Ab Sciex) and data analysis was performed used MetIDQ Oxygen 2976 (Biocrates Life Sciences Innsbruck, Austria). The identified lipids are annotated according to LIPID MAP database (https://www.lipidmaps.org).

### Data analysis

The concentrations of about 500 lipids from CF patients and healthy donors were measured and statistically analyzed by univariate method using GraphPad Prism 8.0. The results were reported according to their mean standard error of the mean (SEM) or their standard deviation (SD). The significant difference was established by performing unpaired t-test with Welch’ s correction in normally distributed datasets or Mann Whitney test in not-normally distributed datasets. The normal distribution was verified according to D'Agostino and Pearson test. Two-stage linear step-up procedure of Benjamini, Krieger and Yekutieli was performed as False Discovery Rate approach (Q = 1%). Volcano plot analysis was carried out using criteria of p-value (p < 0.05) and log2 fold change (FC) larger than ± 0.58 to select differentially lipid abundance in CF patients versus controls^59,60^. Finally, the lipidic dataset was processed according to a multivariate analysis^61,62^ using MetaboAnalyst 4.0 (http://www.metaboanalyst.ca). The lipidic dataset was imputed to remove the missing values, log (10)-transformed and scaled according to the Pareto method. The resulted dataset was used to define PLS-DA (Partial Least Squares—Discriminant Analysis), and VIP (Variable Importance in Projection) score.

## Supplementary Information

Supplementary Figures.

Supplementary Tables.

## Data Availability

The data supporting the findings of this study are available within the paper and its Supplementary Information.

## Abbreviations

CF: Cystic fibrosis
PI: Pancreatic insufficiency
CFTR: Cystic fibrosis transmembrane regulator
FA: Fatty acids
SFA: Saturated fatty acids
MUFA: Monounsaturated fatty acids
PUFA: Polyunsaturated fatty acids
CTRL: Controls
PA: *Pseudomonas Aeruginosa*
DG: Diacylglycerols
TG: Triacylglycerols
LPC: Lysophosphatidylcholine
PCaa: Phosphatidylcholine with acyl–acyl residue
PCae: Phosphatidylcholine with acyl–alkil residue
CE: Cholesterol esters
Cer: Ceramides
SM: Sphingomyelins
AC: Acylcarnitine
HexCer: Hexosylceramides
PLS-DA: Partial least squares-discriminant analysis
VIP: Variable importance in projection
PS: Pancreatic sufficiency
FEV: Forced expiratory volume
