# Supplementary material for: Lipidomic alterations in human saliva from cystic fibrosis patients
Source: Sci Rep. 2023 Jan 12;13:600. doi: 10.1038/s41598-022-24429-6 (PMC9837121; doi:10.1038/s41598-022-24429-6)
Supplement: Supplementary file 1 — Supplementary Figures. [file 41598_2022_24429_MOESM1_ESM.pdf]

Lipidomic alterations in human saliva from Cystic Fibrosis patients

Marianna Caterino [1,2], Roberta Fedele[2], Vincenzo Carnovale[3], Alice Castaldo [3], Monica Gelzo[1,2], Paola Iacotucci[4], Margherita Ruoppolo[1,2]* & Giuseppe Castaldo[1,2]

1. Department of Molecular Medicine and Medical Biotechnology, University of Naples Federico II, Naples, Italy;
2. CEINGE - Biotecnologie Avanzate F. Salvatore, s.c.ar.l., 80145 Napoli, Italy
3. Department of Translational Medical Sciences, University of Naples Federico II, Italy
4. Department of Clinical Medicine and Surgery, University of Naples Federico II, Naples, Italy, Italy

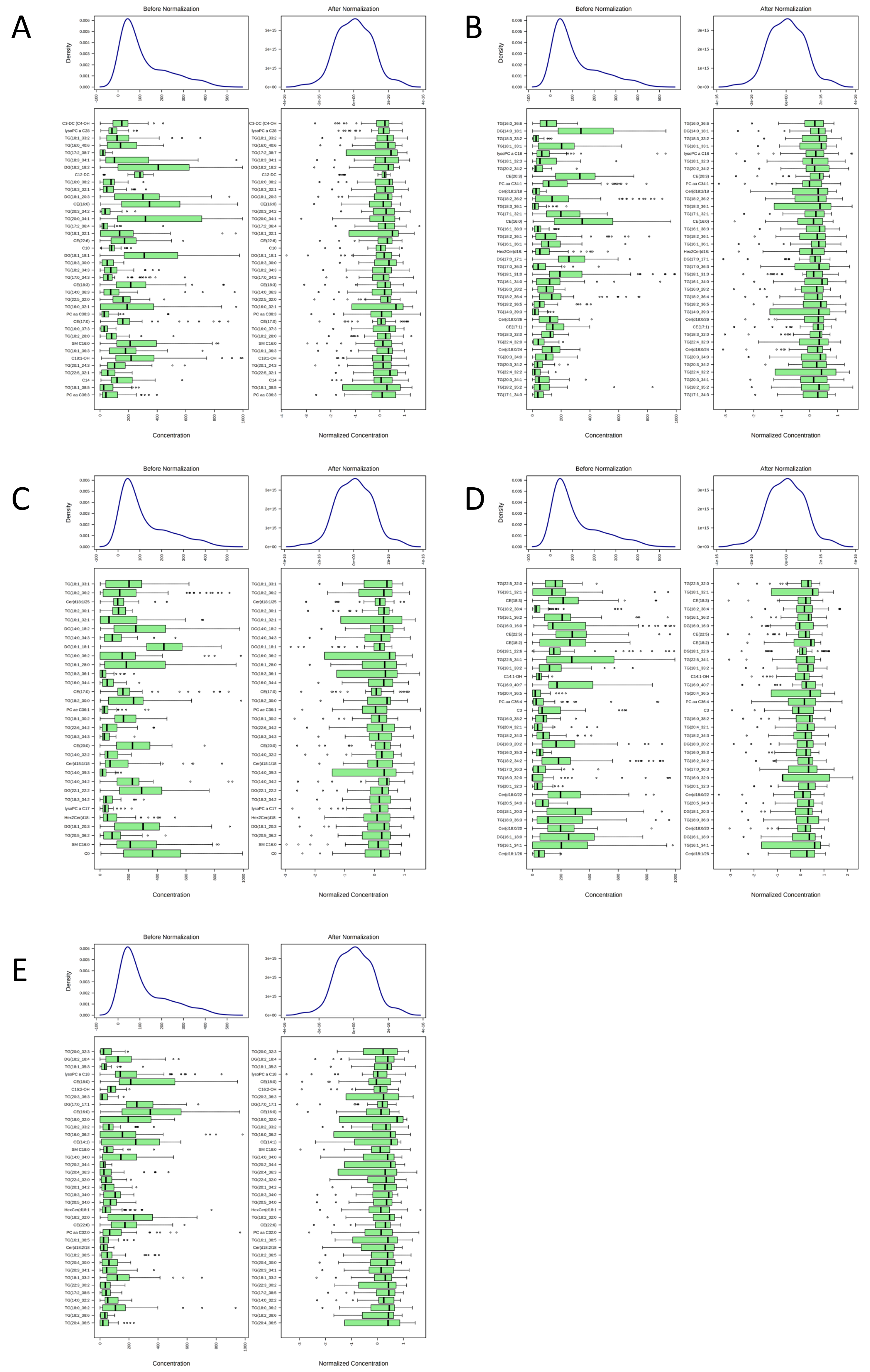

Supplementary Figure 1. Normalized distributions of lipids concentrations from A) CF_notPA and CF_PA; B) CF_P SUF and CF_INS; C) CF_FEV_mild and CF_FEV_severe; D) CF_notLD and CF_LD and E) CF_noRD and CF_RD datasets.

.

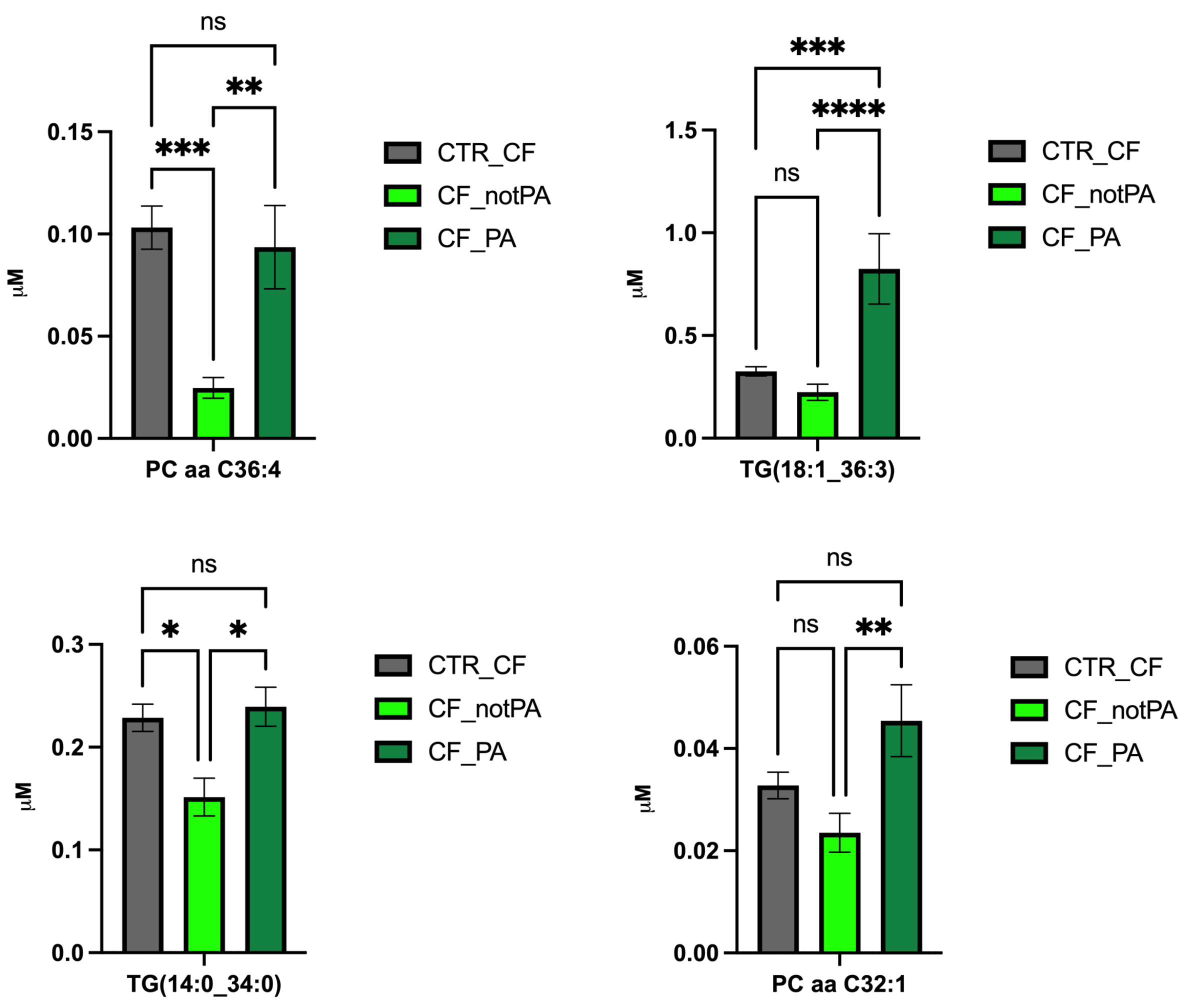

Lipids	Multiple comparisons test	Mean or Mean rank differences	Summary	Adjusted P Value
	CTR_CF vs. CF_notPA	0,07839	***	0,0007
	CTR_CF vs. CF_PA	0,009535	ns	0,8664
PC aa C36:4	CF_notPA vs. CF_PA	-0,06886	**	0,0082
	CTR_CF vs. CF_notPA	0,1016	ns	0,6756
	CTR_CF vs. CF_PA	-0,4992	***	0,0001
TG(18:1_36:3)	CF_notPA vs. CF_PA	-0,6008	****	<0,0001
	CTR_CF vs. CF_notPA	0,00925	ns	0,2732
	CTR_CF vs. CF_PA	-0,01266	ns	0,0825
PC aa C32:1	CF_notPA vs. CF_PA	-0,02191	**	0,0046
	CTR_CF vs. CF_notPA	0,07709	**	0,007
	CTR_CF vs. CF_PA	-0,0108	ns	0,877
TG(14:0_34:0)	CF_notPA vs. CF_PA	-0,08789	**	0,0056

Supplementary Figure 2. Significantly different lipid concentrations in CTR_CF (heathy controls, n=64), CF_notPA (patients without *P. aeruginosa* colonization, n=32) and CF_PA (patients with *P. aeruginosa* colonization, n=38). Plots represents the original lipid concentrations (means ± SD). Multiple comparison was performed by ordinary one-way ANOVA test and Hold-Sidak's multiple comparison test in normally distributed datasets and Kruskal-Wallis test and Dunn's multiple comparison test in not-normally distributed datasets. The normal distribution was verified according to D'Agostino Pearson test. The mean differences between original concentrations and the value of adjusted p-value were reported to each comparison.

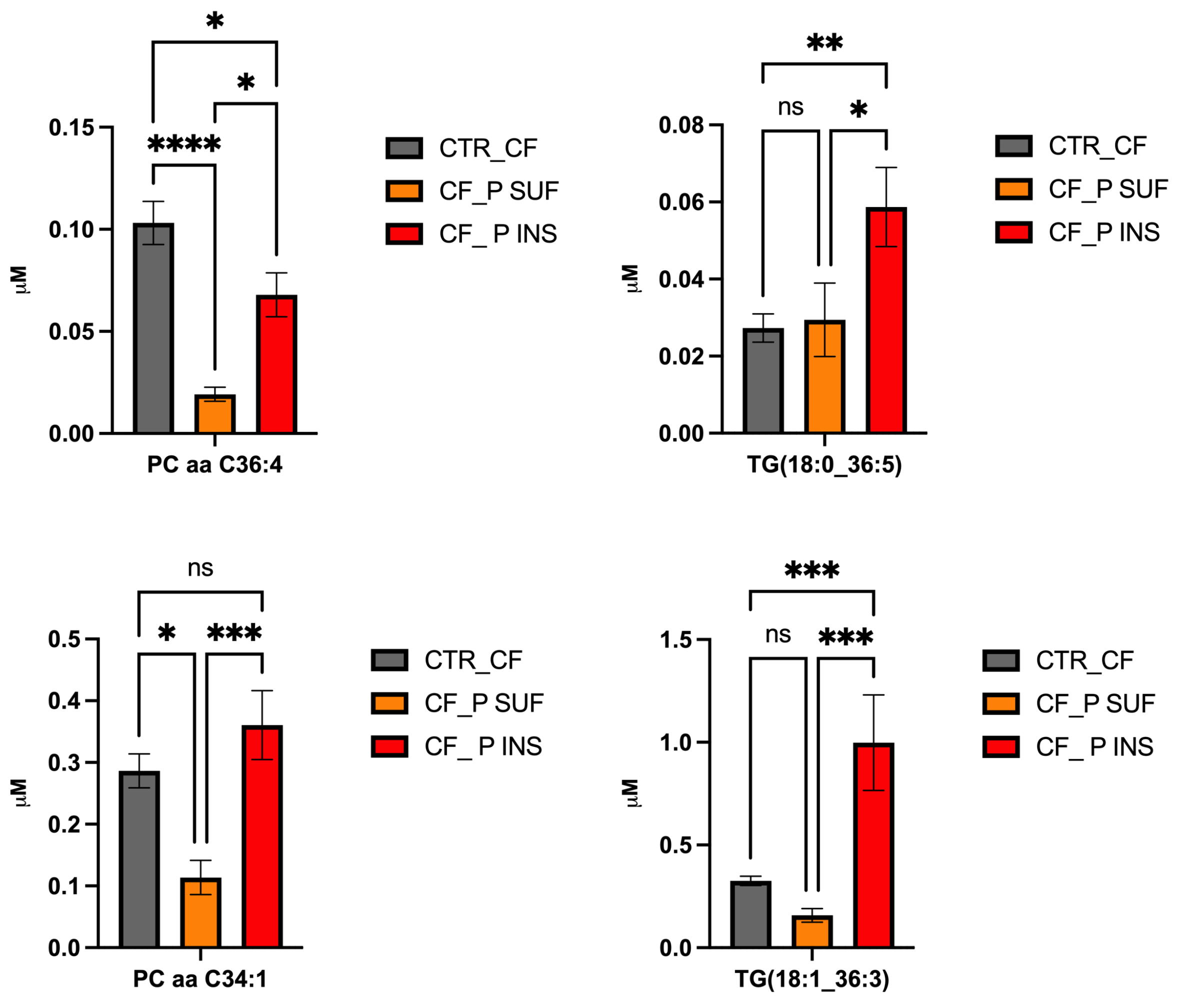

Lipids	Multiple comparisons test	Mean or Mean rank differences	Summary	Adjusted P Value
	CTR_CF vs. CF_SUFF	0,08389	****	<0,0001
	CTR_CF vs. CF_INS	0,03522	*	0,0419
PC aa C36:4	CF_SUFF vs. CF_INS	-0,04868	*	0,0254
	CTR_CF vs. CF_SUFF	-0,002143	ns	0,9795
	CTR_CF vs. CF_INS	-0,03139	**	0,0038
TG(18:0_36:5)	CF_SUFF vs. CF_INS	-0,02925	*	0,0395
	CTR_CF vs. CF_SUFF	0,1727	*	0,0126
	CTR_CF vs. CF_INS	-0,07422	ns	0,3276
PC aa C34:1	CF_SUFF vs. CF_INS	-0,2469	***	0,0006
	CTR_CF vs. CF_SUFF	0,1675	ns	0,7114
	CTR_CF vs. CF_INS	-0,6731	***	0,0007
TG(18:1_36:3)	CF_SUFF vs. CF_INS	-0,8406	***	0,0008

Supplementary Figure 3. Significantly different lipid concentrations in CTR_CF (heathy controls, n=64), CF_P SUF (patients with pancreatic sufficiency, n=28) and CF_P INS (patients with pancreatic insufficiency, n=42). Plots represents the original lipid concentrations (means ± SD). Multiple comparison was performed by ordinary one-way ANOVA test and Hold-Sidak's multiple comparison test in normally distributed datasets and Kruskal-Wallis test and Dunn's multiple comparison test in not-normally distributed datasets. The normal distribution was verified according to D'Agostino Pearson test. The mean differences between original concentrations and the value of adjusted p-value were reported to each comparison.

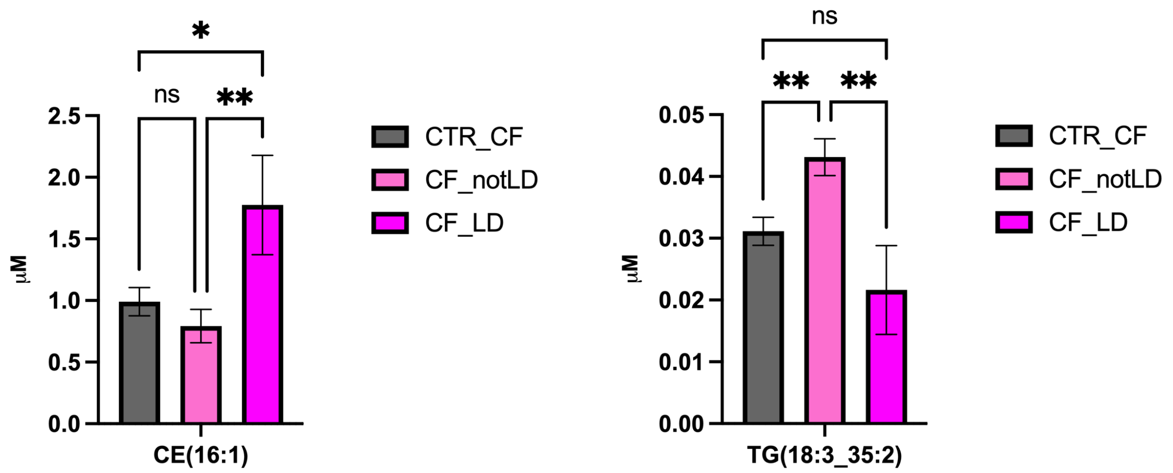

Lipids	Multiple comparisons test	Mean or Mean rank differences	Summary	Adjusted P Value
	CTR_CF vs. CF_notLD	0,1981	ns	0,53
	CTR_CF vs. CF_LD	-0,7846	*	0,0396
CE 16:1	CF_notLD vs. CF_LD	-0,9827	**	0,0074
	CTR_CF vs. CF_notLD	-0,01201	**	0,0061
	CTR_CF vs. CF_LD	0,009493	ns	0,2782
TG(18:3_35:2)	CF_notLD vs. CF_LD	0,0215	**	0,0027

Supplementary Figure 4. Significantly different lipid concentrations in CTR_CF (heathy controls, n=64), CF_notLD (patients without liver disease, n=58) and CF_LD (patients with liver disease, n=12). Plots represents the original lipid concentrations (means ± SD). Multiple comparison was performed by ordinary one-way ANOVA test and Hold-Sidak's multiple comparison test in normally distributed datasets and Kruskal-Wallis test and Dunn's multiple comparison test in not-normally distributed datasets. The normal distribution was verified according to D'Agostino Pearson test. The mean differences between original concentrations and the value of adjusted p-value were reported to each comparison.

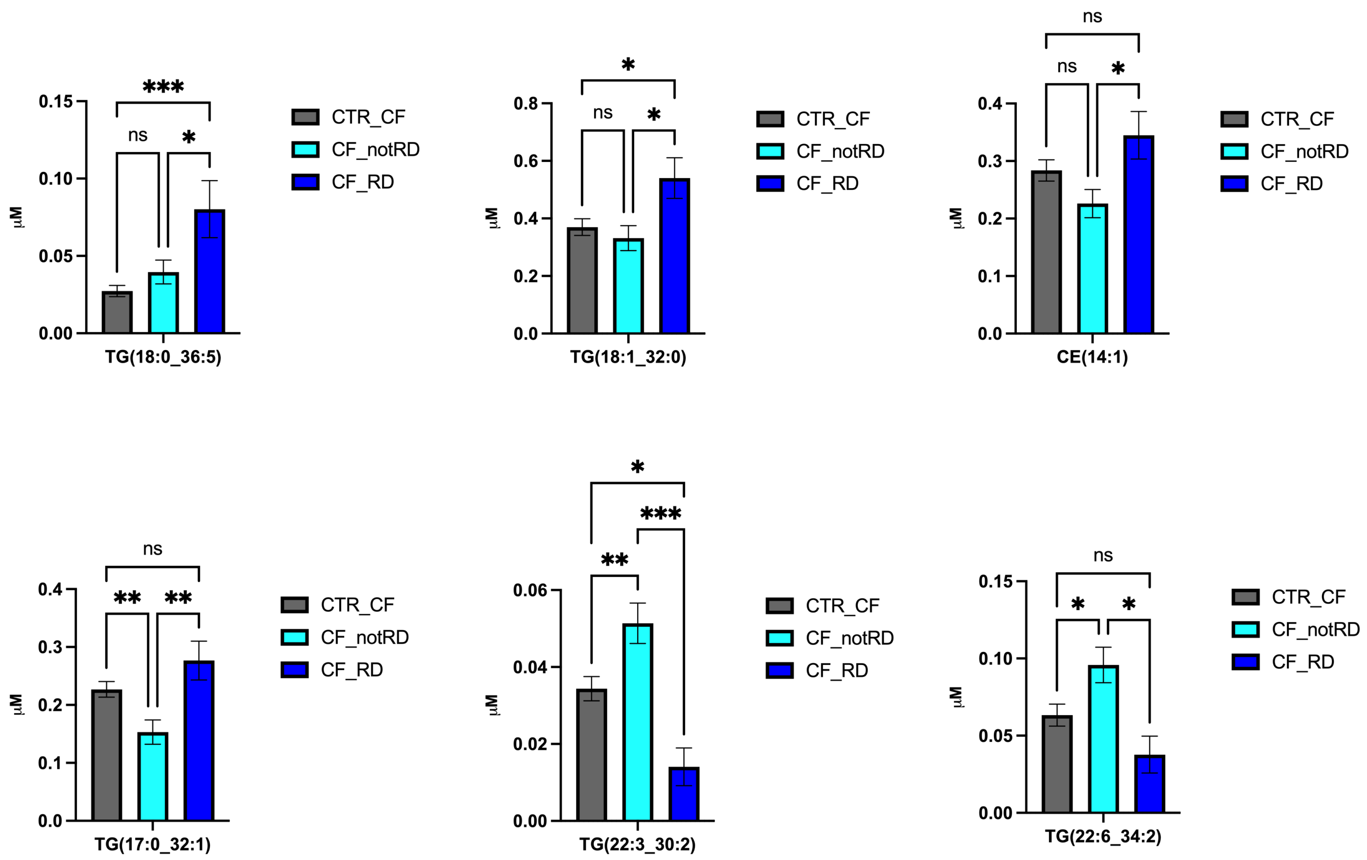

Lipids	Multiple comparisons test	Mean or Mean rank differences	Summary	Adjusted P Value
	CTR_CF vs. CF_notRD	-0,01218	ns	0,3653
	CTR_CF vs. CF_RD	-0,05295	***	0,0004
TG(18:0_36:5)	CF_notRD vs. CF_RD	-0,04077	*	0,01
	CTR_CF vs. CF_notRD	0,03809	ns	0,4558
	CTR_CF vs. CF_RD	-0,1706	*	0,0464
TG(18:1_32:0)	CF_notRD vs. CF_RD	-0,2087	*	0,0222
	CTR_CF vs. CF_notRD	0,05766	ns	0,1397
	CTR_CF vs. CF_RD	-0,06124	ns	0,3734
CE 14:1	CF_notRD vs. CF_RD	-0,1189	*	0,0304
	CTR_CF vs. CF_notRD	0,07378	**	0,008
	CTR_CF vs. CF_RD	-0,0498	ns	0,3658
TG(17:0_32:1)	CF_notRD vs. CF_RD	-0,1236	**	0,0034
	CTR_CF vs. CF_notRD	-0,01701	**	0,0072
	CTR_CF vs. CF_RD	0,02029	*	0,0332
TG(22:3_30:2)	CF_notRD vs. CF_RD	0,03729	***	0,0005
	CTR_CF vs. CF_notRD	-0,03235	*	0,0327
	CTR_CF vs. CF_RD	0,02555	ns	0,4664
TG(22:6_34:2)	CF_notRD vs. CF_RD	0,0579	*	0,025

Supplementary Figure 5. Significantly different lipid concentrations in CTR_CF (heathy controls, n=64), CF_notRD (patients without diabetes, n=54) and CF_RD (patients with diabetes, n=16). Plots represents the original lipid concentrations (means ± SD). Multiple comparison was performed by ordinary one-way ANOVA test and Hold-Sidak's multiple comparison test in normally distributed datasets and Kruskal-Wallis test and Dunn's multiple comparison test in not-normally distributed datasets. The normal distribution was verified according to D'Agostino Pearson test. The mean differences between original concentrations and the value of adjusted p-value were reported to each comparison.
